# Enhancing Patient Experience With Internet Protocol Addressable Digital Light-Emitting Diode Lighting in Imaging Environments: A Phase I Study

**DOI:** 10.2196/11839

**Published:** 2020-06-12

**Authors:** Melanie U Knopp, Katherine Binzel, Chadwick L Wright, Jun Zhang, Michael V Knopp

**Affiliations:** 1 Department of Sports Medicine Seaver College Pepperdine University Malibu, CA United States; 2 Department of Radiology Wright Center of Innovation The Ohio State University Columbus, OH United States; 3 Department of Sport and Health Sciences Technical University of Munich Munich Germany

**Keywords:** ambient lighting, patient comfort, medical imaging, color perception, health care environment, internet protocol–based light-emitting diode lighting

## Abstract

**Background:**

Conventional approaches to improve the quality of clinical patient imaging studies focus predominantly on updating or replacing imaging equipment; however, it is often not considered that patients can also highly influence the diagnostic quality of clinical imaging studies. Patient-specific artifacts can limit the diagnostic image quality, especially when patients are uncomfortable, anxious, or agitated. Imaging facility or environmental conditions can also influence the patient’s comfort and willingness to participate in diagnostic imaging studies, especially when performed in visually unesthetic, anxiety-inducing, and technology-intensive imaging centers. When given the opportunity to change a single aspect of the environmental or imaging facility experience, patients feel much more in control of the otherwise unfamiliar and uncomfortable setting. Incorporating commercial, easily adaptable, ambient lighting products within clinical imaging environments allows patients to individually customize their environment for a more personalized and comfortable experience.

**Objective:**

The aim of this pilot study was to use a customizable colored light-emitting diode (LED) lighting system within a clinical imaging environment and demonstrate the feasibility and initial findings of enabling healthy subjects to customize the ambient lighting and color. Improving the patient experience within clinical imaging environments with patient-preferred ambient lighting and color may improve overall patient comfort, compliance, and participation in the imaging study and indirectly contribute to improving diagnostic image quality.

**Methods:**

We installed consumer-based internet protocol addressable LED lights using the ZigBee standard in different imaging rooms within a clinical imaging environment. We recruited healthy volunteers (n=35) to generate pilot data in order to develop a subsequent clinical trial. The visual perception assessment procedure utilized questionnaires with preprogrammed light/color settings and further assessed how subjects preferred ambient light and color within a clinical imaging setting.

**Results:**

Technical implementation using programmable LED lights was performed without any hardware or electrical modifications to the existing clinical imaging environment. Subject testing revealed substantial variabilities in color perception; however, clear trends in subject color preference were noted. In terms of the color hue of the imaging environment, 43% (15/35) found blue and 31% (11/35) found yellow to be the most relaxing. Conversely, 69% (24/35) found red, 17% (6/35) found yellow, and 11% (4/35) found green to be the least relaxing.

**Conclusions:**

With the majority of subjects indicating that colored lighting within a clinical imaging environment would contribute to an improved patient experience, we predict that enabling patients to customize environmental factors like lighting and color to individual preferences will improve patient comfort and patient satisfaction. Improved patient comfort in clinical imaging environments may also help to minimize patient-specific imaging artifacts that can otherwise limit diagnostic image quality.

**Trial Registration:**

ClinicalTrials.gov NCT03456895; https://clinicaltrials.gov/ct2/show/NCT03456895

## Introduction

To improve the quality of clinical patient imaging studies, imaging environments predominantly consider updating the equipment, and they do not sufficiently appreciate that the quality of an imaging examination is highly influenced by the patient. When there is an unmet need to improve the overall quality of clinical imaging studies, hospitals and imaging centers predominantly focus on updating or replacing the imaging system hardware and rarely consider that patient-specific factors within the imaging environment can also influence overall quality.

When a patient is uncomfortable, anxious, or agitated, any patient motion during the image acquisition can contribute to artifacts that limit the diagnostic quality of the imaging study. This can result in inconclusive results and potentially the need to repeat the imaging study, readminister imaging pharmaceuticals, and re-expose patients to ionizing radiation (eg, radiography, computed tomography [CT], and positron-emission tomography [PET]/CT). Imaging facility or environmental conditions can greatly influence the patient’s experience, comfort, and satisfaction with an imaging study.

By providing patients with the ability to choose aspects of their environmental experience, they feel much more in control of an unfamiliar and uncomfortable setting [1]. Researchers used an experimental audio-visual installation in a PET uptake room and reported reduced patient anxiety during the uptake phase prior to ^18^Fluorine-fluorodeoxyglucose PET imaging [2]. Additionally, studies have reported the use of video goggles as a distraction approach in pediatric patients [3]. The distress experienced in a waiting room was studied, and it set the stage for a more comprehensive approach to address patient comfort and related psychological influences [4]. This study was initiated to enable an in-depth analysis of the perception of light and its psychological influences in order to help develop use case scenarios within imaging environments and provide pilot data for a future prospective clinical trial.

Empirical research indicates that color has a large influence on cognition, affect, and behavior of individuals [5]. Color stimuli consist of multiple dimensions including hue, lightness, and chroma. Hue is defined as the comparability to one of the perceived colors (red, yellow, green, and blue) and is often reported with a hue circle [5]. Lightness is comparable to brightness and is inherently the white to black quality of a color [5]. Chroma resembles saturation and is fundamentally considered the intensity or vividness of the color [6]. Since color is entirely dependent on both photoreceptors and neural processing, it is not a physical quantity, but rather a psychophysical one [7].

In a study associating color and mood, researchers confirmed that color schemes in interior design alone could impact an individual’s mood [8]. Another study suggested that generally warm color schemes increase an individual’s stimulation and muscle tension, whereas cool color schemes tend to relax and decrease tension [9,10]. However, creating one ideal ambiance applicable to all individuals within an environment may be impractical owing to differing individual characteristics and preferences, suggesting that those environments capable of individually modifying color schemes would be most effective [8].

One way to allow for this flexibility in an environment is to use commercial, easily adaptable, ambient lighting products so that patients can personalize their facility environment experience. With such lighting products available, we embarked upon this feasibility demonstration to explore how consumer products in an imaging environment could be used to achieve better patient comfort. The aim of this pilot study was to use a customizable white/colored light-emitting diode (LED) lighting system within a clinical imaging environment and demonstrate the technical feasibility and initial findings of enabling healthy subjects to individualize the ambient lighting and color.

## Methods

### Internet Protocol Addressable Digital Light-Emitting Diode Lighting

Currently, there are different approaches within the consumer LED lighting market. We chose the ZigBee Alliance standard [11] that supports internet protocol (IP) addressable LED lights and interface devices from different manufacturers. Most of these devices provide dedicated software development tools to support broadly utilizable smart devices. Although various manufacturers have such products, we chose the Philips Hue Personal Wireless Lighting system (Signify, Eindhoven, Netherlands) [12]. This system supports uniquely addressable LED lights that can be remotely controlled and individually programmed to different settings of light hue, chroma, and lightness, making such lights fully interoperable and capable of individualized real-time setting adjustments in imaging environments.

**Figure 1:**
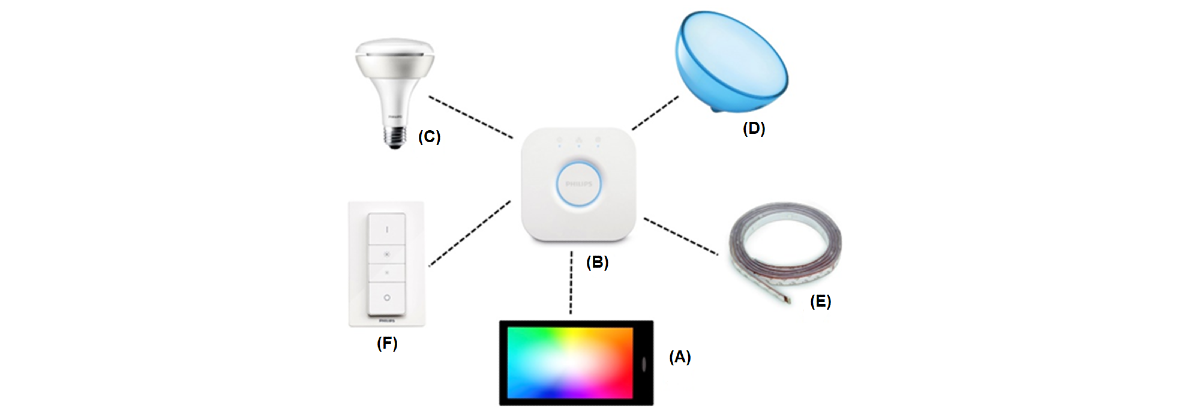
A ZigBee internet protocol (IP)-based lighting system and setup with different styles of Philips Hue white and colored lighting bulbs. (A) A smart device via a Wi-Fi router has access to the hub allowing the Philips Hue app to control light settings in various rooms. The screen of the device shows a Hue circle. Figure adapted [13]. (B) The hub (or bridge) is IP connected and communicates with the lights using the ZigBee 2.4 GHz radiofrequency spectrum multihop mesh network to control light (on/off), intensity, and hue. All lights are powered from normal bulb outlets. Each light functions in the multihop setup in both receive and transmit mode. (C) Light-emitting diode (LED) ZigBee bulbs that can be placed in standard light fixtures. (D) Portable LED wireless Philips Hue Go light. (E) Philips Hue Lightstrips can also be added to enhance the room lighting and ambiance. (F) Philips Hue Dimmer Switch can be used to select from preprogramed light color settings. After the color scene is chosen, this device allows the user to both increase and decrease the dim.

With the lighting system hub in place, we installed BR30 bulbs (Figure 1) [13]. The wirelessly controllable bulbs can be installed in a specific room and then collectively grouped using the unique bulb serial numbers via the Hue smart device app (available on the Apple App Store and Google Play Store). It is also possible to operate multiple hubs on the same network, enabling a well-defined environmental layout with sufficient speed for real-time responsiveness.

### Imaging Facilities

**Figure 2:**
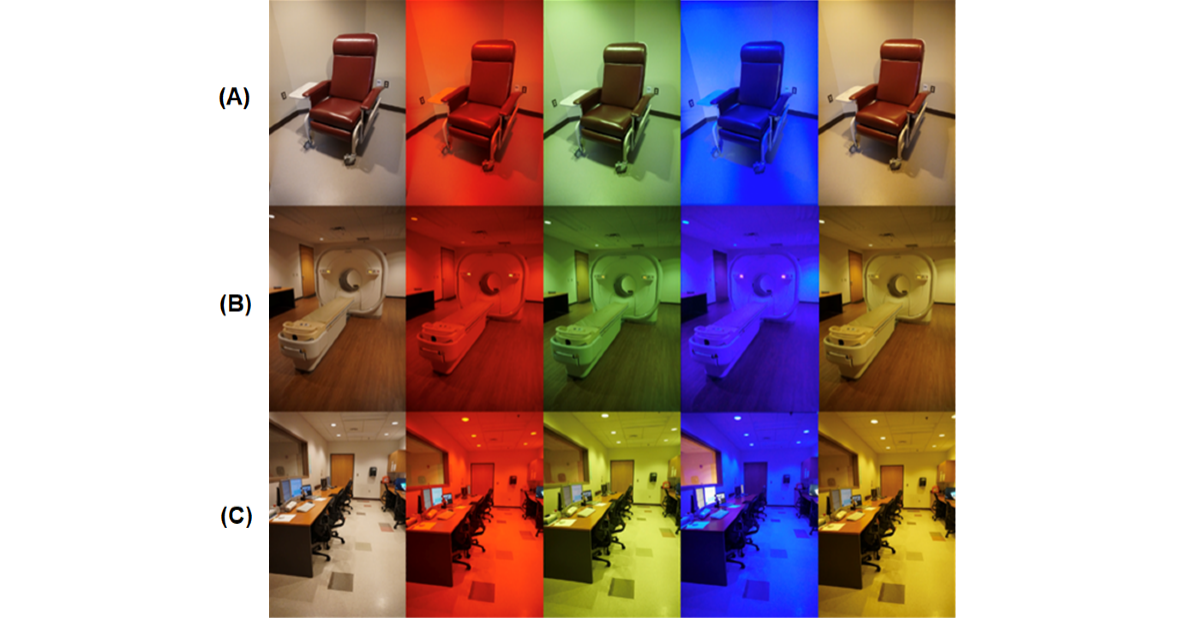
Different color settings in three rooms within an imaging environment. (A) Patient injection/preparation room; (B) scan room of the digital positron-emission tomography/computed tomography (PET/CT) system; and (C) PET/CT control room. In each of these rooms, different color settings are demonstrated as follows: standard bright white light, red light, green light, blue light, and yellow light.

We implemented this IP addressable lighting system in three patient preparation rooms, one PET/CT scan room, and one PET/CT control room (Figure 2). Although specialized app designs can be readily achieved using the software development kit (SDK), we focused on the commercially available product app for ease of adoption by others. With the commercially available product app, different ambiences and scenes can be created by individually or collectively setting the hue, chroma, and lightness for each addressable light in the various rooms. In our experiments, each room was individually addressed, but all lights within each room were collectively setup so that each light in the room produced the same hue, chroma, and lightness within the room.

### Feasibility Trial Population

For this feasibility phase I trial (ClinicalTrials.gov NCT03456895), we enrolled 35 subjects to participate in the patient injection/preparation room experience only. These subjects did not participate in the PET/CT scan room or PET/CT control room experiences. Each subject was coded with a subject ID number and was evaluated inside a patient injection/preparation room while sitting in a standard patient injection chair with the room door closed. There were several steps.

First, a hardcopy questionnaire covering visual impairment, current mood, favorite color, least favorite color, lighting preferences, and preferred color schemes was completed.

Second, an investigator, who was sitting behind the subjects, set the room’s lighting to a full intensity red, identified to the subject as color A. The subjects were exposed to the color A ambience for 30 seconds before being asked to complete another Likert-type scale questionnaire involving five (n=5) or seven (n=30) questions. Upon completion of the color A questionnaire, this procedure was repeated for green light (color B), blue light (color C), and yellow light (color D). The investigator did not name the color but instead used letters to allow the subjects to label the perceived color.

Third, after the four predefined ambient lighting exposures, the subjects completed another survey asking whether they felt that their ability to be productive is influenced by light, which color (A-D) made them feel most relaxed, and which color (A-D) made them feel most anxious.

Fourth, the subjects selected their preferential lighting scheme. The subjects were asked to create their most relaxing light ambience for the room while being able to individually set the hue, chroma, and lightness of the room lights using an app (iOS “Huemote”) within a 3-minute timeframe. After the subjects selected their most relaxing light ambience settings, they completed a final questionnaire asking how calm, uncomfortable, and energetic they felt with the individualized relaxing light ambience. The investigator used the “Color Grab” app to identify the subjects’ chosen color (specifically, the name of the color and its corresponding red, green, blue [RGB] color codes). The entire testing procedure for the subjects was on average 20 minutes.

For this phase I study, only descriptive statistics are reported. A 5-point Likert-type scale was used to assess subjects’ perception with the general layout (1, *strongly disagree*; 2, *disagree*; 3, *neutral*; 4, *agree*; and 5, *strongly agree*). For the charts and overall categorization, we combined in this pilot population all scores of preference involving strong preference and kept the “neutral” answers separate.

## Results

### Characterization of the Subjects

Of the 35 subjects, 17 were male and 18 were female. The average age was 33 years (SD 14 years). Among the 35 subjects, 17 wore prescription glasses and seven used contact lenses. The initial questionnaire data revealed substantial variability in perceptions among subjects, with 71% (25/35) choosing natural light as their preferred lighting preference, followed by 17% (6/35) choosing a warm yellow, 6% (2/35) choosing bright white light, and 6% (2/35) choosing very dim light. It should be noted that bright white light is the most common lighting used in health care environments.

### Red Lighting (Color A)

**Figure 3:**
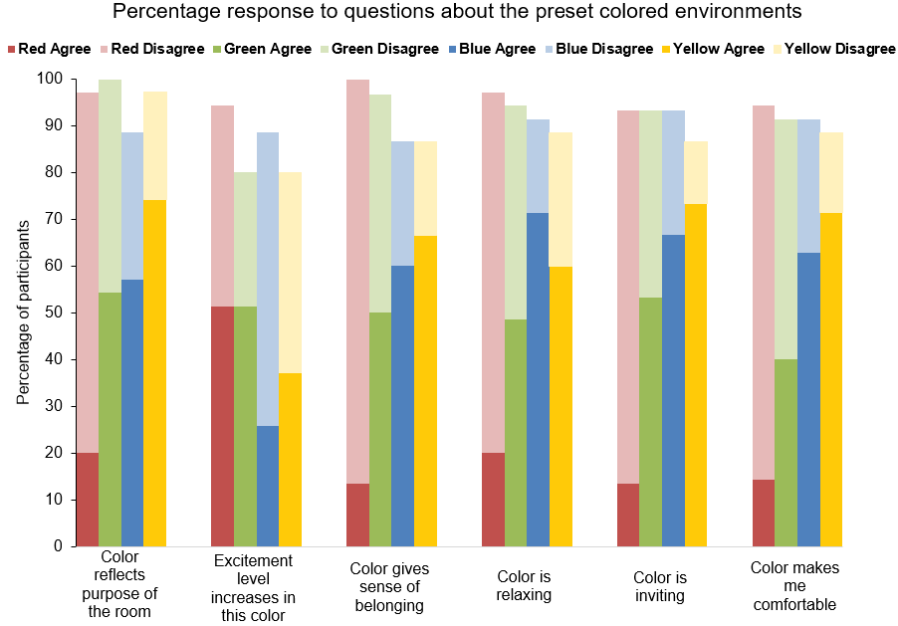
Perceptions related to each color. After experiencing each of the four preset colored environments (red, green, blue, and yellow), subjects completed questionnaires on perceptions related to each color. Bars represent the percentage response distributions for the Likert-type preference questions.

Red was the preferred color for 17% (6/35) of subjects and the least preferred for 3% (1/35). In a subset of 30 subjects, red was classified as a warm color by 80% (24/30) of subjects, but was also associated with an increased excitement level, with less favorable ratings for a sense of belonging, being relaxing, being inviting, and being comforting. Only 20% (7/35) of subjects agreed that red was ideal for the patient preparation room, whereas 77% (27/35) disagreed (Figure 3).

### Green Lighting (Color B)

Green was the preferred color for 14% (5/35) of subjects and the least preferred for 6% (2/35). In a subset of 30 subjects, green was classified as a warm color by 13% of subjects (4/30), with an almost even split between those who perceived green as relaxing, inviting, comforting, and belonging and those who did not. More than half of the subjects (19/35, 54%) indicated that green was ideal for the patient preparation room (Figure 3).

### Blue Lighting (Color C)

Blue was the preferred color for 46% (16/35) of subjects and the least preferred for 6% (2/35). The majority of subjects (22/35, 63%) indicated that blue decreased the excitement level, while increasing the sense of belonging (18/30, 60%), being relaxing (25/35, 71%), being inviting (20/30, 67%), and being comforting (22/35, 63%), despite blue being classified as a warm color by only 13% (4/30). More than half of the subjects (20/35, 57%) reported that blue was ideal for the patient preparation room (Figure 3).

### Yellow Lighting (Color D)

Yellow was never the preferred color among subjects and was the least preferred for 20% (7/35) of subjects. In a subset of 30 subjects, yellow was classified as a warm color by 60% (18/30) of subjects and was associated with a decreased excitement level and other favorable ratings for a sense of belonging, being relaxing, being inviting, and being comforting. A high proportion (26/35, 74%) of subjects indicated that yellow was ideal for the patient preparation room (Figure 3).

### Combined Color Lighting

The most relaxing color for the patient preparation room was blue in 43% (15/35) of subjects, followed by yellow in 31% (11/35), green in 14% (5/35), and red in 11% (4/35). The most anxiety-inducing color was red in 69% (24/35) of subjects, followed by yellow in 17% (6/35), green in 11% (4/35), and blue in 3% (1/35).

### Preferred Lighting

When the subjects individualized their preferred lighting scheme for the patient preparation room and could manually adjust hue, chroma, and lightness, 43% (15/35) preferred blue lighting and 26% (9/35) preferred yellow lighting. The remaining 31% (11/35) of subjects selected a variety of other colors including orange (1/35, 3%), purple (2/35, 6%), red (4/35, 11%), pink (1/35, 3%), and white (3/35, 9%).

### Hardware Costs

A large component of implementing this concept is the organizational setup, as the equipment cost is marginal. A starter kit including a hub and three LED IP addressable A19 bulbs costs approximately US $180/€164. An additional hub costs approximately US $60/€55. Extension bulbs, including BR30 bulbs used in this experiment, cost approximately US $50/€45 each. An additional dimmer switch costs approximately US $25/€23.

This feasibility study established the methodology and demonstrated the technical feasibility to test how individual subjects perceive different colored lighting. The technical implementation using consumer-based commercially available LED lights was realized without any hardware or electrical modifications to the existing imaging environment/facility. The installed IP programmable lights were readily controllable using smartphone or tablet-based apps.

## Discussion

### Principal Findings

This feasibility study demonstrated that a clinical imaging environment can be readily modified to enable an individualized lighting experience with adjustable color schemes using commercially available consumer-based products without specific facility or hardware changes and within manageable costs. The hardware and software used for this study can readily be replaced with alternative technology, making our findings vendor neutral. We have also pilot tested the replacement of hardcopy questionnaires with smartphone or tablet-based surveys that would be even more convenient and efficient for subjects as well as investigators. The subject assessment methodology used in this study can be easily implemented for other clinical applications within health care environments.

Our phase I data indicated that blue lighting was perceived as the most relaxing and was most preferred for the patient preparation room. In contrast, red lighting was perceived as the most anxiety inducing in the patient preparation room. No subject preferred the standard bright white lighting of health care environments, and only 9% (3/35) of subjects preferred a more neutral white lighting for patient preparation rooms when given the opportunity to individualize room lighting.

Available smart device apps can provide patients with individual control of the room’s lighting, allowing them to set their personal preference, which can thereby reduce the stress and anxiety of imaging environments.

### Future Studies

Future clinical studies may further examine the impact of ambient light settings on patient experience, mood, perception, and resting physiology. Color psychology is an essential aspect that may explain the variability among subjects’ perception of different lighting schemes. Color has been proven to have a psychological and physical effect on humans, as explained by the Wright theory of color psychology (Table 1) [14]. It was shown that each hue influences particular psychological modes and can therefore affect the mood and behavior of the individual [15].

The psychological properties of the 11 basic colors are described in Table 2. When the human eye sees light, the different wavelengths lead to different perceptions of light. In the retina, these light waves are transformed into electrical impulses that are processed in the thalamus. According to Angela Wright, these psychological colors relate to body, mind, and emotion [16]. The properties further highlight that individuals perceive color differently. Therefore, to maximize comfort levels, lighting systems need to be versatile and readily adaptable to individual user preferences. Our study confirmed that this is achievable using existing consumer LED IP addressable lighting systems.

Given the small number of subjects in this feasibility trial, we are developing future larger clinical trials that will further assess individualized lighting preferences in clinical imaging environments (ie, patient injection/preparation room, PET/CT scan room, and PET/CT control room) and the impact on clinical patients and healthy subjects. Some aspects of future clinical trials will be the randomized assignment of clinical patients and healthy subjects to rooms with standard bright white lighting versus individualized white/colored lighting and the impact on existing institutional patient satisfaction survey scores, as well as net promoter scores. In addition, these trials in clinical patients will evaluate the rates of patient motion artifacts in PET and CT imaging datasets among PET/CT patients imaged under standard lighting versus individualized white/colored lighting in PET/CT scan rooms to address whether individualized ambient lighting reduces patient motion artifacts in diagnostic imaging studies.

**Table 1:** Seven principal tenants of the Wright theory of color psychology and color harmony [15].

Tenant	Description
1	Each hue affects distinct psychological modes.
2	The psychological effects of color are universal.
3	Every shade, tone, or tint can be classified into one of the four color groups.
4	Every color will harmonize with every other color in the same group.
5	All humanity can be classified into one of four personality types.
6	Each personality type has a natural affinity with one color group.
7	Response to color schemes is influenced by personality type.

**Table 2:** Current psychological interpretation and association of the 11 basic colors [16].

Color	Psychological aspect	Positive effects	Negative effects
Red	Physical	Physical courage, strength, warmth, energy, basic survival, “fight or flight” stimulation, masculinity, and excitement	Defiance, aggression, visual impact, and strain
Blue	Intellectual	Intelligence, communication, trust, efficiency, serenity, duty, logic, coolness, reflection, and calm	Coldness, aloofness, lack of emotion, and unfriendliness
Yellow	Emotional	Optimism, confidence, self-esteem, extraversion, emotional strength, friendliness, and creativity	Irrationality, fear, emotional fragility, depression, anxiety, and suicide
Green	Balance	Harmony, balance, refreshment, universal love, rest, restoration, reassurance, environmental awareness, equilibrium, and peace	Boredom, stagnation, blandness, and enervation
Violet	Spiritual	Spiritual awareness, containment, vision, luxury, authenticity, truth, and quality	Introversion, decadence, suppression, and inferiority
Orange	N/A^a^	Physical comfort, food, warmth, security, sensuality, passion, abundance, and fun	Deprivation, frustration, frivolity, and immaturity
Pink	N/A	Physical tranquility, nurture, warmth, femininity, love, sexuality, and survival of the species	Inhibition, emotional claustrophobia, emasculation, and physical weakness
Grey	N/A	Psychological neutrality	Lack of confidence, dampness, depression, hibernation, and lack of energy
Black	N/A	Sophistication, glamour, security, emotional safety, efficiency, and substance	Oppression, coldness, menace, and heaviness
White	N/A	Hygiene, sterility, clarity, purity, cleanness, simplicity, sophistication, and efficiency	Sterility, coldness, barriers, unfriendliness, and elitism
Brown	N/A	Seriousness, warmth, nature, earthiness, reliability, and support	Lack of humor, heaviness, and lack of sophistication

^a^N/A: not applicable.

### Limitations

A limitation of this phase I trial is that it demonstrates feasibility and findings in healthy subjects with no known diseases in an imaging environment as opposed to patients undergoing imaging procedures for the evaluation of clinical signs or symptoms concerning underlying diseases. Specifically, the healthy subjects in this trial were only tested in the patient preparation rooms, which are used clinically for PET/CT patients, but were not tested in the PET/CT scan room or PET/CT control room. These PET/CT scan room and PET/CT control room evaluations in healthy subjects or clinical patients will be the focus of future studies.

Given that adaptable ambient lighting systems are being incorporated and integrated into many aspects of daily life, we are confident that this work will encourage other researchers to investigate the ambient lighting experience within health care environments in the future.

### Comparison With Prior Work

To date, no prior studies have been reported on this topic and the potential utility within clinical imaging environments.

### Conclusion

This feasibility study demonstrated that individualizing the ambient lighting experience within a clinical imaging environment can be achieved and implemented using commercially available, consumer-grade, IP addressable lighting products. We found variability among the subjects’ perceptions of the various lighting schemes used in this trial, which suggests that an individualized approach to modify the ambient lighting of patient-specific rooms could create more comforting health care environments. This pilot trial found that most subjects (>90%) preferred colored ambient lighting as opposed to standard bright white lighting for patient-specific rooms within the clinical imaging environment.

## Abbreviations

CT: computed tomography
IP: internet protocol
LED: light-emitting diode
PET: positron emission tomography
RGB: red, green, blue
